# Supervisor-subordinate relationships, internal employability, and emotional labor: an exploratory comparative analysis of two supermarket branches in China

**DOI:** 10.3389/fpsyg.2026.1928782

**Published:** 2026-09-11

**Authors:** Yuan Zhang, Ying Yu, Yixin Liu

**Affiliations:** Business School, Zhuhai College of Science and Technology, Zhuhai, Guangdong, China

**Keywords:** attachment theory, emotional labor, internal employability, sociometer theory, supervisor-subordinate relationship

## Abstract

**Introduction:**

This study investigates the relationships among supervisor-subordinate relationship (SSR), internal employability and emotional labor strategies of front-line supermarket employees in China. Drawing on attachment theory and sociometer theory, it examines the indirect role of internal employability and compares the patterns of association across two branch samples.

**Methods:**

A cross-sectional survey was conducted using convenience sampling among 287 front-line supermarket employees in China. Participants comprised 121 front-line employees from one branch of a local privately owned supermarket chain in Shenzhen (Branch A) and 166 front-line employees from one branch of a Western multinational supermarket chain in Beijing (Branch B). Confirmatory factor analyses and measurement-invariance tests were conducted in Mplus. Total, direct and indirect associations were estimated separately for each sample using PROCESS Model 4 with 5,000 bootstrap resamples. Monte Carlo analyses with 20,000 replications were conducted to assess the robustness of the indirect association estimates. An exploratory PROCESS Model 59 analysis was also conducted to examine differences in the estimated associations by sample membership.

**Results:**

In both samples, SSR was positively associated with internal employability (*β* = 0.21 to 0.52, *p* < 0.05) and deep acting (*β* = 0.21 to 0.42, *p* < 0.05) and negatively associated with surface acting (*β* = −0.43 to −0.36, *p* < 0.001). In the Branch B sample, the indirect associations between SSR and both surface acting and deep acting through internal employability were supported. In the Branch A sample, the indirect association with surface acting was not supported, whereas the result for deep acting was supported. The conditional indirect associations for both outcomes were larger in the Branch B sample, although the contextual basis of these differences remains unclear.

**Discussion:**

SSR and internal employability are related but distinct resources in front-line retail work. The indirect association linking SSR to deep acting through internal employability was more consistently supported than the corresponding indirect association involving surface acting. Although the observed patterns varied between the two samples, the present design does not establish that these differences were caused by organizational or institutional conditions. Future research should examine potential contextual influences using longitudinal, multi-source, and multi-site designs.

## 1 Introduction

The retail sector is undergoing substantial change in response to market saturation, digitalization, and the rapid adoption of automated service technologies (Kohli et al., 2025). These developments are particularly relevant to front-line employees, whose work continues to require intensive customer interaction while becoming increasingly exposed to technological substitution and changing skill requirements (Kong et al., 2026). In China, 56% of surveyed retail enterprises reported using public generative artificial intelligence services in 2024, while 36% had developed proprietary platforms and 72% expected these technologies to reduce operating costs [Deloitte China and China Chain Store and Franchise Association (CCFA), 2024]. Such changes may improve operational efficiency, but they may also intensify employees’ concerns about skill relevance, job continuity, and future opportunities within their organizations.

Under these conditions, employees may regard effective emotional regulation as a means of meeting service expectations and demonstrating their continued value to the organization (Ashforth and Humphrey, 1993; Grandey, 2000). Emotional labor refers to employees’ efforts to regulate their feelings and emotional expressions in accordance with organizational display expectations (Hochschild, 1983; Grandey, 2000). Two primary strategies of emotional labor are surface acting, characterized by the simulation of appropriate emotions without internal adjustment, and deep acting, characterized by the effort to genuinely experience the emotions being displayed (Hochschild, 1983; Grandey, 2000).

These strategies are conceptually distinct and may have different implications. Surface acting entails a separation between felt and displayed emotions, often resulting in emotional dissonance and the consumption of energetic resources (Hochschild, 1983; Grandey, 2000). Deep acting involves greater alignment between internal feelings and outward expression and may support more authentic service interactions (Grandey, 2000; Pugh, 2022). Identifying the conditions associated with these strategies is therefore important for both employee well-being and service delivery.

Previous emotional labor research has extensively examined display rules, customer interactions, employee well-being, and service performance (Hochschild, 1983; Grandey, 2000; Hülsheger and Schewe, 2011). However, less attention has been paid to how employees’ relationships with supervisors shape emotional regulation through their perceptions of internal career prospects (Grandey et al., 2020). This omission is important because supervisors influence front-line employees’ work experiences by allocating shifts and assignments, providing feedback, facilitating access to training, and signaling whether employees’ contributions are recognized (Lindblom et al., 2016). Supervisory treatment may therefore influence not only employees’ immediate work experiences but also their judgments about their value and future within the organization.

To examine this relational influence, the present study focuses on supervisor-subordinate relationship (SSR), which captures the interpersonal and affective quality of the relationship between a supervisor and an employee (Law et al., 2000). In the Chinese context, SSR may extend beyond formal task exchange to include personal concern, reciprocity, relational obligation, and relationship-based support (Law et al., 2000; Tsui et al., 2007). Such experiences may shape employees’ access to recognition, information, resources, and developmental opportunities in workplaces where guanxi remains salient (Farh et al., 1998). A high-quality SSR may strengthen employees’ confidence in their organizational standing, whereas a weaker relationship may increase uncertainty about available support and future opportunities (Law et al., 2000; Rhoades and Eisenberger, 2002).

This study proposes internal employability as the career-related link through which SSR may become relevant to emotional labor. Internal employability refers to employees’ perceptions of their ability to retain their current positions or obtain alternative roles within the same organization (Rothwell and Arnold, 2007). It therefore extends beyond the immediate SSR by capturing broader judgments about employees’ future value and mobility within the organization. This distinguishes internal employability from external employability, which concerns perceived opportunities in the wider labor market (Van der Heijde and Van der Heijden, 2006; Rothwell and Arnold, 2007).

Employees perceiving strong internal employability may value emotional investment because they anticipate continued organizational membership or future opportunities. Such perceptions may encourage deep acting and reduce reliance on surface acting. However, the two are distinct strategies that employees may use independently or concurrently (Grandey, 2000; Diefendorff et al., 2005). Thus, this study examines whether their associations with internal employability differ by strategy.

Attachment theory and sociometer theory provide complementary interpretations for these relationships. Attachment theory emphasizes how dependable and responsive relationships shape perceptions of relational security (Bowlby, 1982; Yip et al., 2018). From this perspective, high-quality SSR may make employees’ immediate relational environment appear more predictable and supportive. Sociometer theory complements this perspective by emphasizing how interpersonal cues signal acceptance, inclusion, and relational value within important social groups (Leary et al., 1995). Relational features of SSR, including recognition and personal concern, may consequently inform employees’ perceptions of their value within the organization. These perspectives highlight the security-related and evaluative meanings that employees may derive from the same supervisory relationships, providing a theoretical basis for linking SSR with broader assessments of internal career prospects.

The proposed relationships may not be uniform across workplace settings. Relational practices remain salient in Chinese organizations, although their expression may vary across workplaces (Farh and Cheng, 2000). Retail branches may also differ in their management practices, workforce composition, labor-market conditions, and access to developmental opportunities. Such contextual differences may shape how employees interpret the relational cues embedded in SSR and evaluate their internal career prospects. However, the study did not directly measure these organizational conditions and therefore does not attribute differences in the estimated associations to any specific contextual factor.

To explore possible variation in the estimated associations across the two available samples, the model was estimated separately for two supermarket branches. The branches differed in ownership background, geographic location, workforce composition, and potentially other unobserved characteristics. After measurement comparability was assessed, separate analyses were conducted for the Branch A and Branch B samples to identify sample-specific patterns. The comparison was exploratory rather than a formal test of organizational or institutional moderation, and neither branch should be treated as representative of a broader ownership category or institutional system. The separate analyses were intended to avoid obscuring variation between the two samples and overgeneralizing findings from a single workplace.

Against this background, the study examines internal employability as a career-related link between SSR and emotional labor and considers whether the proposed relationships receive similar or different support across the two organizational samples. It addresses the following research questions:

*RQ1*: How is SSR associated with internal employability among front-line supermarket employees?

*RQ2*: How is SSR indirectly associated with surface acting and deep acting through internal employability?

*RQ3*: What patterns of support for the hypothesized relationships are observed across the Branch A and Branch B samples?

By addressing these questions, the study connects employees’ immediate supervisory experiences with their broader internal career perceptions and emotional regulation in front-line retail work.

## 2 Hypotheses development

### 2.1 The relationship between SSR and internal employability

SSR refers to the interpersonal exchange and relational bond between a supervisor and an employee (Law et al., 2000). Although SSR and leader-member exchange (LMX) both concern dyadic workplace relationships, they differ in their cultural grounding and substantive scope. Developed primarily within Western organizational research, LMX emphasizes professional reciprocity, resource exchange, and role negotiation within the employment relationship (Graen and Uhl-Bien, 1995). SSR is more directly embedded in the Chinese cultural context of guanxi and encompasses task coordination, interpersonal affect, social reciprocity, and relational obligation (Law et al., 2000). It therefore captures employees’ evaluations of their current relationship with a particular supervisor, including personal concern, mutual support, and reciprocal exchange (Tsui et al., 2006, 2007).

Internal employability represents a conceptually different form of evaluation. It refers to employees’ perceptions of their ability to retain their current positions or obtain alternative roles within the same organization (Rothwell and Arnold, 2007). It concerns perceived skill relevance, employment continuity, adaptability, and mobility within the organization rather than employment opportunities in the wider labor market (Van der Heijde and Van der Heijden, 2006).

SSR and internal employability are therefore related but distinct constructs. SSR concerns the current quality of an employee’s relationship with a specific supervisor, whereas internal employability reflects a broader and future-oriented assessment of the employee’s continued value and mobility within the organization. A supportive supervisory relationship does not necessarily imply strong internal employability. Employees may receive favorable interpersonal treatment while remaining uncertain about their qualifications, job continuity, or access to internal opportunities. Conversely, employees may perceive strong internal employability because of their experience, qualifications, scarce expertise, or organizational demand even when their relationship with a particular supervisor is less favorable.

Although SSR does not constitute an assessment of employability, the quality of SSR may provide employees with career-related information when they evaluate their organizational future. Within higher-quality SSR, employees may receive developmental feedback, access to assignments, recommendations, and information about internal opportunities (Kraimer et al., 2011; Wu and Parker, 2017; Yang et al., 2018). Higher-quality SSR may also facilitate feedback seeking and relationship building (Beenen et al., 2017). These relational and developmental cues may inform employees’ judgments about whether guidance, recognition, and developmental opportunities are likely to remain available. Thus, although SSR is conceptually distinct from internal employability, it may serve as one source of information shaping employees’ perceptions of their internal career prospects.

Attachment theory and sociometer theory offer complementary perspectives on how employees may interpret these supervisory experiences. These theories are used to explain the possible meanings employees derive from the quality of the supervisory relationship rather than to redefine SSR or propose two separately measured psychological mechanisms. Attachment theory emphasizes how individuals interpret the dependability, responsiveness, and consistency of important relational environments (Bowlby, 1982; Yip et al., 2018). From this perspective, higher-quality SSR may signal that guidance and assistance are likely to remain available when work-related difficulties arise.

Sociometer theory emphasizes how interpersonal cues inform individuals’ perceptions of acceptance, inclusion, and relational value within important social groups (Leary et al., 1995). Supervisory recognition and personalized support may therefore be interpreted as one source of information about an employee’s standing in the workplace (Rhoades and Eisenberger, 2002). However, such relational cues do not determine internal employability, which also depends on employees’ competencies, experience, occupational demand, and access to internal opportunities.

The two theories consequently highlight complementary meanings that employees may derive from supervisory experiences. Attachment theory draws attention to perceived relational dependability, whereas sociometer theory focuses on perceived acceptance and relational value. These perspectives suggest that SSR may carry both security-related and evaluative meaning, helping employees interpret what the relationship signals about their broader internal career prospects.

Accordingly, higher-quality SSR is expected to be associated with stronger perceptions of internal employability, whereas lower-quality SSR may convey fewer positive relational and developmental cues concerning employees’ organizational future. The following hypothesis is proposed:

*H1*: SSR is positively associated with internal employability.

### 2.2 The relationship between SSR and emotional labor strategies

Emotional labor refers to employees’ regulation of feelings and emotional expressions to meet the emotional requirements of their work roles (Hochschild, 1983). In retail settings, employees commonly rely on surface acting and deep acting to satisfy organizational display expectations. Surface acting regulates outward expressions without changing internal states, whereas deep acting seeks to generate genuine feelings that match required emotional display (Grandey, 2003; Grandey et al., 2004).

The quality of SSR may be relevant to these strategies because supervisors form an important part of the immediate interpersonal environment in which employees perform emotional work (Liu et al., 2023). Front-line retail employees frequently encounter demanding customers, unpredictable interactions, intensive interpersonal contact, and pressure to maintain appropriate emotional displays. Supervisors may influence how employees respond to these demands by providing guidance, practical assistance, recognition, and emotional support (Zhang et al., 2026). The perceived availability of such support may shape whether employees engage more fully with their emotional roles or rely primarily on outward compliance.

Attachment theory provides one explanation for the association between SSR and emotional labor. Higher-quality SSR, characterized by dependability and responsiveness, may increase employees’ confidence that guidance and assistance will be available during difficult service encounters and reduce their perceived need for interpersonal self-protection (Foster et al., 2007). Employees who regard their relationship with their supervisor as reliable and understanding may feel more able to engage with the emotional demands of their roles (Brotheridge and Lee, 2002; Mostafa et al., 2025).

Sociometer theory offers a complementary interpretation by emphasizing the evaluative meaning of relational cues embedded in SSR. Recognition, constructive feedback, personal concern, and reciprocal exchange may signal employees’ acceptance and relational value within the workplace (Leary, 2005; Perinelli et al., 2022). Collectively, these perspectives suggest that SSR may carry both security-related and evaluative meanings that shape how employees approach the emotional demands of front-line service work.

These interpretations suggest that higher-quality SSR may provide more favorable relational conditions for deep acting. Employees who perceive their supervisory relationship as dependable and supportive may be more willing to invest the cognitive and emotional effort required to modify their underlying feelings (Grandey, 2000). They may reinterpret difficult encounters, consider customers’ perspectives, or generate emotions that are more consistent with service expectations rather than modifying only their outward expressions. The recognition and relational value conveyed through SSR may further strengthen employees’ willingness to engage more genuinely with their service roles. Accordingly, SSR is expected to be positively associated with deep acting.

Lower-quality SSR may, by contrast, be associated with greater reliance on surface acting. Limited relational support, inconsistent interaction, weak trust, or interpersonal distance may increase employees’ uncertainty about whether guidance or assistance will be available during emotionally demanding encounters (Brotheridge and Lee, 2002). Employees may therefore maintain greater psychological distance from their roles and focus on satisfying formal emotional display requirements. Surface acting enables employees to modify their outward expressions without changing their underlying feelings (Grandey, 2000), providing an immediate means of meeting service expectations when the supervisory relationship is perceived as less dependable.

Thus, SSR is expected to show contrasting associations with the two strategies. Higher-quality SSR is expected to be associated with greater deep acting and lower surface acting, whereas lower-quality SSR may be associated with outward emotional compliance without a corresponding change in experienced emotions. This leads to the following hypotheses:

*H2a*: SSR is negatively associated with surface acting.

*H2b*: SSR is positively associated with deep acting.

### 2.3 The relationship between internal employability and emotional labor strategies

Internal employability is theoretically expected to be associated with emotional labor. According to sociometer theory, employees continuously monitor cues that inform them about their interpersonal value and social acceptance within the organization (Leary et al., 1995). Perceptions of internal employability can serve as such cues because they signal whether employees are regarded as valued members who are likely to remain included in the organization and retain access to future opportunities (Baranchenko et al., 2020). When employees believe that they can retain their current positions or move into alternative internal roles, they are more likely to infer that they are socially accepted and organizationally valued (Baranchenko et al., 2020). By contrast, weaker internal employability may signal uncertainty about future inclusion and, by implication, a diminished sense of social worth, which may in turn shape how employees regulate their emotions in service interactions. In this sense, internal employability can be understood as a socially meaningful cue that informs employees’ emotional regulation strategies.

This interpretation based on sociometer theory helps explain why internal employability may be positively associated with deep acting. Deep acting requires employees to regulate not only their outward expressions but also their internal feelings, which involves greater cognitive and emotional effort than mere surface compliance (Grandey, 2000). Employees who perceive stronger internal employability may be more willing to invest in this form of regulation. Anticipated continued organizational membership may strengthen their motivation to maintain valued relationships and perform their work roles effectively. When employees perceive themselves as accepted and valued, they are more likely to engage in emotionally authentic regulation that sustains their standing within the organization. Accordingly, stronger internal employability should encourage deep acting.

Internal employability may also shape employees’ reliance on surface acting. Stronger perceptions of internal employability may encourage emotional investment, whereas weaker perceptions may reduce the perceived value of such investment. Employees who perceive limited internal career prospects may be less inclined to expend emotional effort in their service roles, particularly when continued organizational membership and future opportunities appear uncertain. Under these circumstances, surface acting may offer a less demanding means of complying with emotional display expectations while requiring minimal personal engagement (Leary et al., 1995; Van den Broeck et al., 2014; Baranchenko et al., 2020). On this basis, the following hypotheses are proposed:

*H3a*: Internal employability is negatively associated with surface acting.

*H3b*: Internal employability is positively associated with deep acting.

### 2.4 The indirect role of internal employability

The preceding arguments suggest that internal employability may provide a career-related link between SSR and emotional labor strategies. Experiences embedded in the current supervisory relationship may provide relational, evaluative, and developmental cues that employees incorporate into broader, future-oriented assessments of their organizational prospects. Internal employability captures this broader career appraisal rather than the immediate quality of the supervisory relationship itself.

In the first stage of the proposed indirect relationship, SSR is expected to be positively associated with internal employability. Employees with higher-quality supervisory relationships may have greater access to developmental feedback, valued assignments, recommendations, and information about internal opportunities. These experiences may provide relevant information when employees assess whether their competencies are recognized and whether guidance, support, and developmental resources are likely to remain available. However, SSR represents only one potential source of such information. Internal employability may also reflect employees’ qualifications, occupational experience, skill relevance, organizational demand, and the availability of internal positions.

Attachment theory and sociometer theory provide complementary interpretive perspectives on how the quality of SSR may acquire career-related meaning. From an attachment perspective, higher-quality SSR, characterized by consistency and responsiveness, may signal relational dependability and reduce uncertainty about whether work-related guidance and assistance will remain available under demanding conditions. From a sociometer perspective, relational cues embedded in SSR, including recognition, feedback, and access to developmental opportunities, may inform employees’ perceptions of their acceptance, value, and standing within the workplace. Employees may combine these security-related and evaluative cues with information about their competencies, job requirements, and organizational opportunities when assessing their prospects for continued employment and internal mobility.

Internal employability therefore represents a broader career appraisal rather than a direct measure of attachment security or perceived organizational standing. The proposed cognitive process is that high-quality SSR may provide employees with cues about relational dependability and organizational value, which may then be incorporated into their assessments of future employment continuity, skill relevance, and internal mobility. This appraisal may influence the perceived value employees attach to continued cognitive and emotional investment in their service roles.

In the second stage, employees who anticipate continued organizational membership or realistic internal opportunities may perceive greater value in investing cognitive and emotional effort in their work roles. Such employees may be more willing to engage in deep acting because aligning experienced and displayed emotions requires sustained effort. By contrast, employees who perceive limited internal prospects may be less inclined to make this investment and may rely more heavily on surface acting to satisfy emotional display requirements while maintaining psychological distance.

These proposed associations should be considered separately because surface acting and deep acting are distinct emotional labor strategies rather than opposite ends of a single continuum (Grandey, 2000; Diefendorff et al., 2005). Employees may use either strategy, or both strategies, across different service interactions. Accordingly, a positive association between internal employability and deep acting does not, by itself, imply an equivalent negative association with surface acting. The relationship of internal employability with each strategy therefore requires separate empirical examination.

This reasoning suggests that SSR may be indirectly associated with emotional labor through internal employability. Higher-quality SSR may provide relational, evaluative, and developmental information associated with stronger perceptions of internal career continuity and mobility. Stronger internal employability may, in turn, be associated with greater deep acting and lower surface acting. Given the cross-sectional design, these proposed relationships are examined as statistical indirect associations rather than established temporal or causal mechanisms. Accordingly, the following hypotheses are proposed:

*H4a*: SSR is indirectly and negatively associated with surface acting through internal employability.

*H4b*: SSR is indirectly and positively associated with deep acting through internal employability.

**Figure 1:**
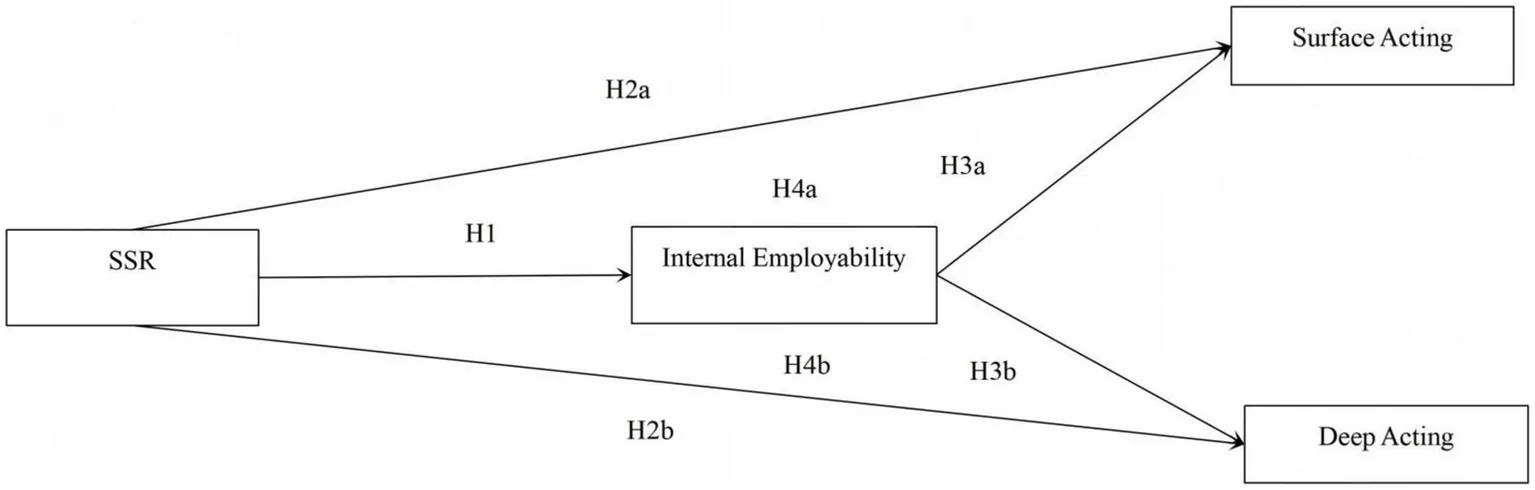
Theoretical model. SSR, supervisor-subordinate relationship.

Figure 1 presents the conceptual model.

## 3 Methodology

### 3.1 Participants and procedure

Based on a comparative two-site design, a cross-sectional questionnaire survey was conducted. The final sample included 287 front-line employees, including cashiers, customer service staff, and deli associates, drawn from two large organizations in the Chinese retail sector. Convenience sampling was employed, with data collected from two supermarket branches. Branch A was a store in Shenzhen, a Tier 1 city in southern China, belonging to a large local privately owned supermarket chain. All 121 front-line employees at the branch were invited and completed the questionnaire, yielding a 100% response rate. Branch B was a store in Beijing, a Tier 1 city in northern China, operated by a Western multinational supermarket chain. Of the 180 questionnaires distributed to front-line employees at this branch, 166 were completed and returned, yielding a response rate of 92.22%.

The two branches were selected as they provided access to workplace samples differing in ownership background, geographic location, and workforce composition. Branch A represented a local private retail setting, whereas Branch B represented a Western multinational retail setting. However, the two branches were not treated as pure representatives of contrasting institutional systems. They also differed in geographic location, workforce composition, and potentially other unmeasured organizational characteristics. Thus, the comparison was exploratory and focused on the pattern of hypothesis support within each sample rather than on isolating a causal effect of ownership or institutional context.

Data were collected through a mobile-based electronic survey platform. A manager at each participating branch acted as an internal coordinator and distributed the survey link through the branch’s internal communication channels. The managers did not have access to individual responses.

Before completing the survey, employees were informed that participation was voluntary and that the data would be used solely for academic research. Participants were assured that their responses were anonymous and confidential, and that neither their supervisors nor the employer would have access to the data. No personally identifiable information, including names, telephone numbers, or employee identification numbers, was collected. Although managers distributed the survey link, they were not informed about which employees chose to participate, and participation or non-participation had no employment-related consequences. The questionnaire was organized into separate sections and used previously validated measures to reduce evaluation apprehension, item priming, and respondent fatigue.

Table 1 summarizes the demographic profiles of the two branch samples. Participants in the Branch A sample were slightly older than those in the Branch B sample (mean = 33.85 vs. 32.49 years) and had substantially longer organizational tenure (mean = 7.34 vs. 4.03 years). Full distributions by gender, education, and marital status are reported in the table.

**Table 1:** Sample description.

Variable	Category	Branch A		Branch B	
		Frequency	Percentage (%)	Frequency	Percentage (%)
Gender	Male	35	28.93	78	46.99
	Female	86	71.07	88	53.01
Age (years)	≤30	50	41.32	60	36.14
	31–40	41	33.88	98	59.04
	≥41	30	24.80	8	4.82
Education	Junior high school and below	12	9.92	0	0.00
	Senior high school	51	42.15	5	3.01
	College	29	23.97	62	37.35
	Bachelor’s degree	25	20.66	96	57.83
	Master’s degree and above	4	3.30	3	1.81
Marital status	Single	46	38.02	46	27.71
	Married	75	61.98	120	72.29
Tenure (years)	≤5	55	45.45	126	75.90
	6–10	28	23.14	38	22.89
	11–15	28	23.14	2	1.21
	16–20	7	5.79	0	0
	≥21	3	2.48	0	0

For the Branch A sample, *N* = 121. For the Branch B sample, *N* = 166.

### 3.2 Measures

All scales, originally published in English, were carefully translated and back-translated following established procedures to maintain both linguistic and conceptual equivalence (Brislin, 1970). The final questionnaire was administered in Chinese. Items were rated on a five-point Likert scale, with response options ranging from 1 (“strongly disagree”) to 5 (“strongly agree”). To provide complementary evidence of internal consistency, both Cronbach’s alpha (*α*) and McDonald’s omega (*ω*) were calculated for all measures in both samples. McDonald’s omega was derived from the standardized factor loadings and residual variances estimated in the confirmatory factor analysis (CFA). Compared with Cronbach’s alpha, omega does not impose the assumption of tau-equivalence and allows factor loadings to vary across indicators (McDonald, 1999; Dunn et al., 2014; Hayes and Coutts, 2020).

#### 3.2.1 SSR

The quality of SSR was measured using five items from the scale developed by Law et al. (2000), which assess the degree of mutual trust and supportive interactions between supervisors and employees. Examples include “During holidays or after office hours, I would call my supervisor or visit him/her” and “When there are conflicting opinions, I will definitely stand on my supervisor’s side.” The scale demonstrated high internal consistency in the Branch A sample with a Cronbach’s alpha (*α*) of 0.94 and McDonald’s omega (*ω*) of 0.94. For the Branch B sample, Cronbach’s alpha was 0.76 and McDonald’s omega was 0.77, indicating acceptable but comparatively modest internal consistency (Nunnally and Bernstein, 1994; Dunn et al., 2014; Hayes and Coutts, 2020).

#### 3.2.2 Internal employability

Internal employability was assessed using the 6-item scale developed by Rothwell and Arnold (2007). This measure captures employees’ perceptions of their value and career development potential within their current organization. Example items include “I am confident that I could find another job in this organization that matches my professional background” and “I have a good chance of being promoted in this organization.” The scale demonstrated excellent reliability for the Branch A sample (*α* = 0.94, *ω* = 0.94). In the Branch B sample, both Cronbach’s alpha and McDonald’s omega were 0.79, indicating acceptable but comparatively modest internal consistency (Nunnally and Bernstein, 1994; Dunn et al., 2014; Hayes and Coutts, 2020).

#### 3.2.3 Emotional labor

Emotional labor was measured using Diefendorff et al.’s (2005) 11-item multidimensional scale, which differentiates between the two key strategies. Surface acting (comprising 7 items) assesses the intentional faking or suppression of emotions (e.g., “Fake a good mood when interacting with customers”). Deep acting (comprising 4 items) evaluates the effort to genuinely modify internal feelings to match organizational display rules (e.g., “Try to actually experience the emotions that I must show to customers”). For surface acting, Cronbach’s alpha and McDonald’s omega were both 0.95 in the Branch A sample. In the Branch B sample, Cronbach’s alpha was 0.78 and McDonald’s omega was 0.79. For deep acting, Cronbach’s alpha and McDonald’s omega were both 0.93 in the Branch A sample. In the Branch B sample, Cronbach’s alpha was 0.79 and McDonald’s omega was 0.80. These estimates indicate high internal consistency in the Branch A sample and acceptable, although comparatively modest, internal consistency in the Branch B sample (Nunnally and Bernstein, 1994; Dunn et al., 2014; Hayes and Coutts, 2020).

As shown in Table 2, omega closely corresponded to the Cronbach’s alpha estimates, providing broadly consistent internal-consistency estimates. Across the measures in the Branch B sample, McDonald’s omega coefficients ranged from 0.77 to 0.80. Although these coefficients fall within commonly accepted ranges, they indicate lower score precision than the corresponding measures in the Branch A sample (Nunnally and Bernstein, 1994; Dunn et al., 2014; Hayes and Coutts, 2020). Because the PROCESS analyses relied on observed composite scores, measurement error was not explicitly accounted for and may have affected the magnitude and precision of the estimated regression coefficients and indirect associations. Accordingly, estimates derived from the Branch B sample were interpreted with appropriate caution. Measurement invariance was examined separately, although its establishment does not eliminate between-sample differences in reliability or score precision.

**Table 2:** Reliability estimates for the key variables.

Variable	Number of items	Branch A *α*	Branch A *ω*	Branch B *α*	Branch B *ω*
SSR	5	0.94	0.94	0.76	0.77
Internal employability	6	0.94	0.94	0.79	0.79
Surface acting	7	0.95	0.95	0.78	0.79
Deep acting	4	0.93	0.93	0.79	0.80

*α*, Cronbach’s alpha; *ω*, McDonald’s omega.

#### 3.2.4 Control variables

Gender, age, education, marital status, and organizational tenure were included as control variables because they may be associated with the focal variables and emotional labor outcomes (Grandey and Gabriel, 2015; Van der Heijde and Van der Heijden, 2006).

### 3.3 Sample size and power analysis

Using G^*^Power 3.1 (Faul et al., 2009), the minimum detectable effect sizes were estimated based on the available sample sizes, an alpha level of 0.05, a target power of 0.80 (Cohen, 1988), and seven predictors in the most complex regression equation. The minimum detectable overall regression effect sizes were approximately *f^2^* = 0.13 for the Branch A sample and *f^2^* = 0.09 for the Branch B sample. Both values were below the benchmark of *f^2^* = 0.15 for a medium effect (Cohen, 1988), indicating that both samples had adequate power to detect medium overall regression effects. However, these estimates do not indicate the power to detect individual regression coefficients, indirect associations, or interaction terms in the exploratory Model 59 analyses.

### 3.4 Analytical strategy

The analyses proceeded in four stages. Mplus 7.0 was used for confirmatory factor and measurement-invariance analyses, and SPSS PROCESS version 4.1 was used for regression-based analyses. The CFA and measurement invariance analyses were conducted using robust maximum likelihood estimation (MLR).

First, CFAs were conducted separately for the two samples to assess the factorial structure and discriminant validity of SSR, internal employability, surface acting, and deep acting. The hypothesized four-factor model was compared with three alternative models combining theoretically related constructs or loading all items onto a single factor.

Second, multi-group CFAs (MG-CFAs) assessed measurement comparability across the samples. Configural, metric, and scalar invariance were tested sequentially by imposing equality constraints on factor loadings and item intercepts. Changes in CFI, RMSEA, and SRMR were evaluated using established recommendations (Cheung and Rensvold, 2002; Chen, 2007; Putnick and Bornstein, 2016).

Third, total, direct and indirect associations were estimated separately for each sample using PROCESS Model 4 (Hayes, 2022). The indirect associations of SSR with surface acting and deep acting through internal employability were tested using 5,000 bootstrap resamples and 95% confidence intervals (CIs). Control variables were included as covariates. Given the cross-sectional design, the estimates were interpreted as statistical indirect associations rather than causal mediation.

As a sensitivity check within this stage, a Monte Carlo sensitivity analysis was conducted with 20,000 replications. It generated CIs for the indirect effects based on the estimated sampling distributions of the two relevant regression coefficients. Monte Carlo and PROCESS bootstrap CIs were compared as a sensitivity check.

Finally, PROCESS Model 59 was used to examine whether the estimated direct and indirect relationships differed by sample membership. Because no directional moderation hypotheses were proposed and sample membership reflected multiple organizational and demographic differences, these results were interpreted as exploratory sample comparisons rather than causal moderation by ownership or institutional context.

## 4 Results

### 4.1 Common method variance assessment

Self-reported questionnaires were used to collect all variables from the same participants at a single time point. Therefore, there was a risk that common method variance (CMV) could inflate the observed relationships. The procedural remedies implemented to mitigate this risk, including anonymity assurances, the omission of personally identifiable information, and the use of validated scales with clearly separated sections, have been described in Section 3.1 (Participants and procedure).

As a preliminary statistical assessment, Harman’s single-factor test was performed separately for the samples (Podsakoff et al., 2003). For the Branch A sample, the Kaiser-Meyer-Olkin (KMO) measure of sampling adequacy was 0.91. Bartlett’s test of sphericity was also significant (*χ^2^* = 2369.69, *df* = 231, *p* < 0.001) (Kaiser, 1970). Harman’s one-factor analysis indicated that the first unrotated factor accounted for 40.99% of the total variance. For the Branch B sample, the KMO measure was 0.88, and Bartlett’s test of sphericity was significant (*χ^2^* = 1301.88, *df* = 231, *p* < 0.001) (Kaiser, 1970). The first unrotated factor explained 32.66% of the total variance. The variance explained by a single factor was well below the 50% threshold in both datasets (Podsakoff et al., 2003).

Although neither first factor accounted for more than half of the total variance, the 50% threshold represents only a conventional rule and should not be treated as a rigorous statistical criterion for establishing the absence of CMV (Podsakoff et al., 2012). Moreover, the proportions of variance explained by the first factor, particularly the 40.99% observed in the Branch A sample, are not negligible in a self-report study. These results therefore do not justify dismissing CMV as a potential concern.

As reported in Section 4.2, the hypothesized four-factor model fitted the data substantially better than the one-factor model in both samples, indicating that the measures were not adequately represented by a single general factor. Nevertheless, neither the CFA model comparison nor Harman’s single-factor test can identify or quantify all forms of method-related covariance or rule out social desirability bias. These tests therefore provide only partial evidence regarding CMV rather than definitive proof that method bias was absent.

### 4.2 Confirmatory factor analysis

To assess the discriminant validity of the key constructs, CFAs were performed separately for the two branch samples using Mplus 7.0. The hypothesized four-factor model (SSR, internal employability, surface acting, and deep acting) was compared with alternative models: a three-factor model combining surface acting and deep acting into a single factor, a three-factor model combining SSR and internal employability, a two-factor model merging SSR with internal employability and surface acting with deep acting, and a one-factor model loading all items onto a single factor.

For the Branch A sample (*N* = 121), Table 3 shows that the hypothesized four-factor model demonstrated excellent fit (*χ^2^* = 235.07, *df* = 203, *χ^2^*/*df* = 1.16, RMSEA = 0.04, CFI = 0.98, TLI = 0.98, SRMR = 0.05) (Hu and Bentler, 1999). All alternative models exhibited substantially poorer fit. In particular, combining SSR and internal employability into a single factor resulted in markedly worse fit (*χ^2^* = 700.91, *df* = 206, *χ^2^*/*df* = 3.40, RMSEA = 0.14, CFI = 0.76, TLI = 0.73, and SRMR = 0.15).

**Table 3:** Confirmatory factor analysis results for the Branch A sample.

Model	*χ* ^2^	*df*	*χ*^2^/*df*	RMSEA	CFI	TLI	SRMR
Four-factor model	235.07	203	1.16	0.04	0.98	0.98	0.05
Three-factor model (SA + DA)	573.56	206	2.78	0.12	0.82	0.80	0.13
Three-factor model (SSR + IE)	700.91	206	3.40	0.14	0.76	0.73	0.15
Two-factor model (SSR + IE, SA + DA)	1033.98	208	4.97	0.18	0.60	0.56	0.19
One-factor model	1393.65	209	6.67	0.22	0.43	0.37	0.20

*N* = 121. *χ*^2^ values are robust maximum likelihood estimate (MLR) scaled chi-square statistics. df, degrees of freedom. RMSEA, root mean square error of approximation; CFI, Comparative Fit Index; TLI, Tucker-Lewis Index, SRMR, standardized root mean square residual. SA, surface acting; DA, deep acting; IE, internal employability.

As presented in Table 4, for the Branch B sample (*N* = 166), the hypothesized four-factor model demonstrated acceptable overall fit to the data (*χ^2^* = 280.54, *df* = 203, *χ^2^/df* = 1.38, RMSEA = 0.05, CFI = 0.94, TLI = 0.93, SRMR = 0.05). Values of CFI and TLI above 0.90 are commonly considered indicative of acceptable fit, whereas values approaching 0.95 indicate a more stringent level of fit (Hair et al., 2010; Hu and Bentler, 1999). The alternative models generally fitted the data less well. The three-factor model combining SSR and internal employability produced (*χ^2^* = 363.56, *df* = 206, *χ^2^*/*df* = 1.76, RMSEA = 0.07, CFI = 0.87, TLI = 0.85, and SRMR = 0.06). Although some absolute fit indices remained acceptable, its incremental fit indices were notably weaker than those of the hypothesized four-factor model.

**Table 4:** Confirmatory factor analysis results for the Branch B sample.

Model	*χ* ^2^	*df*	*χ*^2^/*df*	RMSEA	CFI	TLI	SRMR
Four-factor model	280.54	203	1.38	0.05	0.94	0.93	0.05
Three-factor model (SA + DA)	582.79	206	2.82	0.10	0.69	0.66	0.17
Three-factor model (SSR + IE)	363.56	206	1.76	0.07	0.87	0.85	0.06
Two-factor model (SSR + IE, SA + DA)	450.27	208	2.16	0.08	0.80	0.78	0.07
One-factor model	472.71	209	2.26	0.09	0.78	0.76	0.08

*N* = 166. *χ*^2^ values are MLR scaled chi-square statistics. df, degrees of freedom. RMSEA, root mean square error of approximation; CFI, Comparative Fit Index; TLI, Tucker-Lewis Index; SRMR, standardized root mean square residual. SA, surface acting; DA, deep acting; IE, internal employability.

Overall, the hypothesized four-factor model provided the best fit in both branch samples. In particular, the poorer fit of the models combining SSR and internal employability supports the empirical distinction between these two constructs. All factor loadings were statistically significant (*p* < 0.001), and most exceeded the recommended threshold of 0.50 (Hair et al., 2010). In the Branch B sample, the loading of the third SSR item and that of the second surface-acting item were both 0.49. These items were retained because their loadings were close to the commonly used threshold and their removal might have compromised the content validity of the measures (Tabachnick and Fidell, 2007). Thus, the findings support the discriminant validity of SSR, internal employability, surface acting, and deep acting in both branch samples. The acceptable fit of the four-factor models also provided a basis for subsequent measurement-invariance testing.

### 4.3 Measurement invariance across the two samples

A sequence of MG-CFAs was conducted in Mplus 7.0 to assess configural, metric, and scalar measurement invariance across the two samples (Cheung and Rensvold, 2002; Chen, 2007; Putnick and Bornstein, 2016). The model comparisons were evaluated using changes in CFI, RMSEA, and SRMR rather than relying solely on the chi-square difference test, which is sensitive to sample size. The results of the measurement invariance tests are presented in Table 5.

**Table 5:** Results of measurement invariance tests across the two branch samples.

Model	*χ* ^2^	*df*	CFI	TLI	RMSEA	SRMR	ΔCFI	ΔRMSEA	ΔSRMR
Configural	513.21	406	0.968	0.964	0.043	0.050	—	—	—
Metric	551.77	424	0.962	0.958	0.046	0.065	−0.006	0.003	0.015
Scalar	565.47	442	0.963	0.961	0.044	0.070	0.001	−0.002	0.005

*N* = 287. Changes in fit indices for the metric model were calculated relative to the configural model, whereas changes for the scalar model were calculated relative to the metric model. df, degrees of freedom. CFI, comparative fit index; TLI, Tucker–Lewis index; RMSEA, root mean square error of approximation; SRMR, standardized root mean square residual. TLI is reported as an indicator of overall model fit, whereas measurement-invariance decisions were based on the more commonly recommended changes in CFI, RMSEA, and SRMR. CFI, TLI, RMSEA, SRMR, and the reported change indices are presented to three decimal places to preserve the small differences used in evaluating invariance and to avoid rounding that could obscure their relation to the recommended cutoff criteria. Because the MLR scaling correction is calculated separately for each model, the chi-square statistic for the multi-group model is not expected to equal the sum of the chi-square statistics from the two separately estimated models.

The configural invariance model specified the same four-factor structure in both samples while allowing the factor loadings and item intercepts to be estimated freely. The model demonstrated acceptable fit, *χ*^2^(406) = 513.21, CFI = 0.968, TLI = 0.964, RMSEA = 0.043, and SRMR = 0.050. This result indicates that the same basic pattern of item-to-factor relationships was tenable across the two samples.

Metric invariance was then examined by constraining the corresponding factor loadings to equality across the two groups. The metric model also demonstrated acceptable fit, *χ*^2^(424) = 551.77, CFI = 0.962, TLI = 0.958, RMSEA = 0.046, and SRMR = 0.065. Relative to the configural model, the changes in fit were ΔCFI = −0.006, ΔRMSEA = 0.003, and ΔSRMR = 0.015. These changes remained within the recommended criteria of an absolute change in CFI no greater than 0.010, an increase in RMSEA no greater than 0.015, and an increase in SRMR no greater than 0.030 for metric invariance (Cheung and Rensvold, 2002; Chen, 2007). Metric invariance was therefore supported.

Scalar invariance was subsequently tested by additionally constraining the corresponding item intercepts to equality across groups. The scalar model demonstrated acceptable fit, *χ*^2^(442) = 565.47, CFI = 0.963, TLI = 0.961, RMSEA = 0.044, and SRMR = 0.070. Compared with the metric model, the changes in fit were ΔCFI = 0.001, ΔRMSEA = −0.002, and ΔSRMR = 0.005. These values remained within the recommended criteria for scalar invariance, including an absolute change in CFI no greater than 0.010, an increase in RMSEA no greater than 0.015, and an increase in SRMR no greater than 0.010 (Chen, 2007).

The configural, metric, and scalar invariance results indicate that the four constructs were measured in a sufficiently comparable manner across the two samples. This finding supports the interpretation of sample-specific structural relationships using a common measurement framework. It does not, by itself, establish that the corresponding structural paths or indirect effects are equal across the samples.

### 4.4 Demographic description and correlation analysis

Tables 6 and 7 summarize the means, standard deviations, and Pearson correlation coefficients for the Branch A and Branch B samples, respectively.

**Table 6:** Means, standard deviations, and correlations for the Branch A sample.

Variable	Mean	SD	1	2	3	4	5	6	7	8	9
Gender	1.71	0.46	-								
Age	33.85	7.96	0.23^*^	-							
Education	2.65	1.02	−0.27^**^	−0.25^**^	-						
Marital status	1.62	0.49	0.21^*^	0.63^**^	−0.23^**^	-					
Organizational tenure	7.34	5.66	0.15	0.83^**^	−0.19^*^	0.54^**^	-				
SSR	2.32	1.07	−0.15	−0.25^**^	0.20^*^	−0.20^*^	−0.28^**^	(0.94)			
Internal employability	2.17	0.89	−0.08	−0.42^**^	0.14	−0.27^**^	−0.42^**^	0.32^**^	(0.94)		
Surface acting	3.85	0.94	0.03	0.23^*^	−0.12	0.08	0.26^**^	−0.46^**^	−0.33^**^	(0.95)	
Deep acting	2.42	1.03	−0.15	−0.26^**^	0.32^**^	−0.10	−0.26^**^	0.30^**^	0.37^**^	−0.28^**^	(0.93)

*N* = 121. ^*^ *p* < 0.05, ^**^ *p* < 0.01. SSR, supervisor-subordinate relationship. Figures in parentheses indicate Cronbach’s *α* reliability coefficients. Gender was coded 1 = male and 2 = female; education was coded from 1 = junior high school and below to 5 = master’s degree and above; marital status was coded 1 = single and 2 = married.

**Table 7:** Means, standard deviations, and correlations for the Branch B sample.

Variable	Mean	SD	1	2	3	4	5	6	7	8	9
Gender	1.53	0.50	-								
Age	32.49	4.37	−0.15	-							
Education	3.58	0.58	−0.09	0.01	-						
Marital status	1.72	0.45	0.04	0.64^**^	−0.03	-					
Organizational tenure	4.03	2.12	−0.10	0.50^**^	−0.08	0.38^**^	-				
SSR	3.91	0.67	0.07	−0.02	0.07	0.11	0.05	(0.76)			
Internal employability	4.05	0.64	0.06	0.04	0.09	0.09	0.11	0.53^**^	(0.79)		
Surface acting	2.02	0.57	−0.06	0.03	0.06	−0.09	−0.06	−0.37^**^	−0.65^**^	(0.78)	
Deep acting	3.99	0.75	0.09	0.02	−0.02	0.11	0.12	0.44^**^	0.64^**^	−0.47^**^	(0.79)

*N* = 166. ^*^ *p* < 0.05, ^**^ *p* < 0.01. SSR, supervisor-subordinate relationship. Figures in parentheses indicate Cronbach’s *α* reliability coefficients. Gender was coded 1 = male and 2 = female; education was coded from 1 = junior high school and below to 5 = master’s degree and above; marital status was coded 1 = single and 2 = married.

#### 4.4.1 Demographic variables

The associations involving demographic factors differed across the two branch samples. In particular, age and organizational tenure were significantly and negatively associated with key constructs in the Branch A sample (Table 6). Age was negatively correlated with SSR (*r* = −0.25, *p* < 0.01) and internal employability (*r* = −0.42, *p* < 0.01). Similarly, organizational tenure was negatively associated with SSR (*r* = −0.28, *p* < 0.01) and internal employability (*r* = −0.42, *p* < 0.01).

In contrast, demographic characteristics were not significantly associated with the main study variables in the Branch B sample (Table 7). Neither age nor organizational tenure was significantly correlated with SSR (*r* = −0.02, *p* > 0.05; *r* = 0.05, *p* > 0.05) or internal employability (*r* = 0.04, *p* > 0.05; *r* = 0.11, *p* > 0.05). Similarly, other control variables, such as gender and education, were not significantly related to any focal constructs. Overall, these results indicate sample-specific differences in the observed demographic associations and should not be generalized beyond the two branch samples.

#### 4.4.2 Key study variables

The bivariate associations among the main constructs were directionally consistent across the two samples (Tables 6, 7), offering initial support for the study’s hypotheses.

Higher-quality SSR was linked to greater internal employability in both samples, with correlations of *r* = 0.32 (*p* < 0.01) in the Branch A sample and *r* = 0.53 (*p* < 0.01) in the Branch B sample. SSR was also negatively associated with surface acting (Branch A: *r* = −0.46; Branch B: *r* = −0.37, *p* < 0.01) and positively associated with deep acting (Branch A: *r* = 0.30; Branch B: *r* = 0.44, *p* < 0.01).

Similarly, internal employability showed a strong relationship with emotional labor strategies. It was negatively associated with surface acting (Branch A: *r* = −0.33; Branch B: *r* = −0.65, *p* < 0.01) and positively associated with deep acting (Branch A: *r* = 0.37; Branch B: *r* = 0.64, *p* < 0.01). In summary, the correlations involving internal employability were descriptively larger in the Branch B sample, although formal differences between the correlations were not tested.

### 4.5 Hypothesis testing

Hypotheses (H1-H4) were examined using the PROCESS macro for SPSS (Model 4, version 4.1) with 5,000 bootstrap resamples (Hayes, 2022). The analyses were performed separately for the Branch A and Branch B samples. Gender, age, education, marital status, and organizational tenure were included as control variables.

#### 4.5.1 Indirect effect analyses for the branch A sample

The indirect effect analyses provided mixed support for the hypothesized indirect associations in the Branch A sample (Tables 8, 9). The regression analysis confirmed that SSR was significantly and positively associated with internal employability (*b* = 0.18, *β* = 0.21, *p* < 0.05), supporting H1. Subsequently, before internal employability was entered into the model, SSR was negatively associated with surface acting (*b* = −0.38, *β* = −0.43, *p* < 0.001) and positively associated with deep acting (*b* = 0.20, *β* = 0.21, *p* < 0.05), supporting H2a and H2b.

**Table 8:** Regression analysis results for the Branch A sample.

Variables	Model 1 (internal employability)	Model 2 (surface acting)	Model 3 (surface acting with internal employability)	Model 4 (deep acting)	Model 5 (deep acting with internal employability)
Controls					
Gender	0.06 (0.17)	−0.11 (0.18)	−0.10 (0.18)	−0.08 (0.20)	−0.10 (0.20)
Age	−0.03 (0.02)	0.01 (0.02)	0.01 (0.02)	−0.02 (0.02)	−0.01 (0.02)
Education	0.02 (0.08)	−0.03 (0.08)	−0.03 (0.08)	0.25^**^ (0.09)	0.25^**^(0.09)
Marital status	0.01 (0.20)	−0.26 (0.20)	−0.25 (0.20)	0.32 (0.23)	0.32 (0.22)
Organizational tenure	−0.02 (0.02)	0.02 (0.02)	0.02 (0.02)	−0.03 (0.03)	−0.02 (0.03)
Main effects					
SSR	0.18^*^ (0.07)	−0.38^***^ (0.08)	−0.34^***^ (0.08)	0.20^*^ (0.09)	0.15 (0.09)
Internal employability			−0.18 (0.10)		0.30^**^ (0.11)
*R* ^2^	0.24	0.24	0.27	0.20	0.25
*F*	5.86^***^	6.16^***^	5.91^***^	4.80^***^	5.50^***^

*N* = 121. ^*^ *p* < 0.05, ^**^ *p* < 0.01, ^***^ *p* < 0.001. Unstandardized coefficients are reported with standard errors in parentheses. SSR, supervisor-subordinate relationship. Gender was coded 1 = male and 2 = female; education was coded from 1 = junior high school and below to 5 = master’s degree and above; marital status was coded 1 = single and 2 = married. Collinearity diagnostics indicated no serious multicollinearity; all tolerance values were above 0.20 and all VIF values were below 5.00.

**Table 9:** Estimated indirect associations of SSR with emotional labor through internal employability.

Path	Sample	Estimate	BootSE	95% Bootstrap CI	
				LLCI	ULCI
SSR → internal employability → surface acting	Branch A	−0.03	0.03	−0.10	0.003
	Branch B	−0.28	0.09	−0.43	−0.10
SSR → internal employability → deep acting	Branch A	0.05	0.04	0.001	0.14
	Branch B	0.33	0.10	0.11	0.51

SSR, supervisor-subordinate relationship. CI, confidence interval; LLCI, lower limit of confidence interval; ULCI, upper limit of confidence interval; BootSE, bootstrap standard error.

Internal employability showed a positive association with deep acting (*b* = 0.30, *β* = 0.26, *p* < 0.01), supporting H3b. However, its relationship with surface acting was negative but not statistically significant (*b* = −0.18, *β* = −0.17, *p* = 0.06). H3a was therefore not supported in the Branch A sample.

Finally, the bootstrapped indirect-effect analysis provided a more nuanced assessment of the hypothesized indirect associations between SSR and emotional labor through internal employability (Table 9). The indirect association between SSR and surface acting through internal employability was non-significant, as the 95% CI included zero (Effect = −0.03, BootSE = 0.03, 95% CI [−0.10, 0.003]). Thus, H4a was not supported in the Branch A sample. In contrast, a significant indirect effect was observed for deep acting (Effect = 0.05, BootSE = 0.04, 95% CI [0.001, 0.14]), supporting H4b.

#### 4.5.2 Indirect effect analyses for the branch B sample

Compared with the Branch A sample, the indirect-effect analyses for the Branch B sample provided more consistent support for the hypothesized indirect associations (Tables 10, 9). Consistent with H1, SSR was positively associated with internal employability (*b* = 0.49, *β* = 0.52, *p* < 0.001). SSR was also negatively associated with surface acting (*b* = −0.31, *β* = −0.36, *p* < 0.001) and positively associated with deep acting (*b* = 0.47, *β* = 0.42, *p* < 0.001), thus supporting H2a and H2b. Furthermore, internal employability in the Branch B sample was negatively associated with surface acting (*b* = −0.57, *β* = −0.63, *p* < 0.001) and positively associated with deep acting (*b* = 0.67, *β* = 0.57, *p* < 0.001). This provided support for H3a and H3b. The bootstrapped indirect effects for the Branch B sample were significant for both surface acting (Effect = −0.28, BootSE = 0.09, 95% CI [−0.43, −0.10]) and deep acting (Effect = 0.33, BootSE = 0.10, 95% CI [0.11, 0.51]). These results supported H4a and H4b in the Branch B sample.

**Table 10:** Regression analysis results for the Branch B sample.

Variables	Model 6 (internal employability)	Model 7 (surface acting)	Model 8 (surface acting with internal employability)	Model 9 (deep acting)	Model 10 (deep acting with internal employability)
Controls					
Gender	0.06 (0.09)	−0.02 (0.09)	0.01 (0.07)	0.08 (0.11)	0.04 (0.09)
Age	0.00 (0.01)	0.01 (0.01)	0.01 (0.01)	−0.01 (0.02)	−0.01 (0.01)
Education	0.07 (0.07)	0.07 (0.07)	0.11 (0.06)	−0.05 (0.09)	−0.09 (0.08)
Marital status	−0.01 (0.13)	−0.11 (0.12)	−0.12 (0.10)	0.09 (0.16)	0.10 (0.13)
Organizational tenure	0.03 (0.02)	−0.01 (0.02)	0.00 (0.02)	0.04 (0.03)	0.02 (0.02)
Main effects					
SSR	0.49^***^ (0.06)	−0.31^***^ (0.06)	−0.03 (0.06)	0.47^***^ (0.08)	0.14 (0.08)
Internal employability			−0.57^***^ (0.06)		0.67^***^ (0.08)
*R* ^2^	0.29	0.16	0.44	0.21	0.44
*F*	10.89^***^	4.88^***^	17.77^***^	6.92^***^	17.63^***^

*N* = 166. ^*^ *p* < 0.05, ^**^ *p* < 0.01, ^***^ *p* < 0.001. Unstandardized coefficients are reported with standard errors in parentheses. SSR, supervisor-subordinate relationship. Gender was coded 1 = male and 2 = female; education was coded from 1 = junior high school and below to 5 = master’s degree and above; marital status was coded 1 = single and 2 = married. Collinearity diagnostics indicated no serious multicollinearity; all tolerance values were above 0.20 and all VIF values were below 5.00.

### 4.6 Supplemental Monte Carlo analysis of the indirect associations

As a supplementary sensitivity analysis, the indirect associations of SSR with emotional labor through internal employability were estimated using Monte Carlo simulation in R version 4.4.1 (R Core Team, 2024). The analyses controlled for gender, age, education, marital status, and organizational tenure, consistent with the PROCESS models. Separate regression models were estimated for each branch and outcome using lavaan (Rosseel, 2012). MASS (Venables and Ripley, 2002) generated 20,000 joint draws from the estimated coefficients linking SSR to internal employability and internal employability to emotional labor. The haven package (Wickham et al., 2024) imported the SPSS data file. The product of the two coefficients was calculated for each draw. Percentile-based 95% Monte Carlo confidence intervals were then derived. Results are presented in Table 11.

**Table 11:** Monte Carlo confidence intervals for indirect associations through internal employability.

Outcome	Branch	Indirect association	95% Monte Carlo CI	Interpretation
Surface acting	Branch A	−0.03	[−0.08, 0.001]	Not supported
Surface acting	Branch B	−0.28	[−0.37, −0.19]	Supported, negative
Deep acting	Branch A	0.05	[0.007, 0.12]	Supported, positive
Deep acting	Branch B	0.33	[0.22, 0.45]	Supported, positive

Monte Carlo confidence intervals were based on 20,000 joint simulations of the relevant regression coefficients. Values are reported to two decimal places, except non-zero values with an absolute magnitude below 0.01, which are reported to three decimal places. The Monte Carlo analyses used 121 complete cases for Branch A and 166 complete cases for Branch B.

The Monte Carlo results were consistent with the PROCESS bootstrap results. For surface acting, the indirect association through internal employability was negative and supported in Branch B. In Branch A, the corresponding indirect association was not supported. For deep acting, the indirect associations were positive and supported in both samples.

Overall, the Monte Carlo analysis provided supplementary support for the PROCESS results. The findings indicate differences in the estimated associations across the two branch samples. Given the cross-sectional design, these results should be interpreted as associations rather than evidence of causal or temporal processes.

### 4.7 Supplementary exploratory comparison analysis by sample membership

To conduct a supplementary exploratory analysis, the two samples were pooled, and branch membership was tested as a moderator using PROCESS Model 59 (Hayes, 2022; 1 = Branch A, 2 = Branch B). Gender, age, education, marital status, and organizational tenure were included as control variables. This analysis examined whether the estimated associations differed between branches. However, because branch membership was confounded with organizational, regional, and demographic differences, the analysis could not identify the specific contextual factors underlying any observed variation. The Model 59 findings are supplementary and do not replace the primary hypothesis tests reported in Section 4.5. The pooled and separate sample analyses use different model specifications. Therefore, their estimates are not expected to be numerically identical.

#### 4.7.1 Surface acting as the outcome

In the conditional process analysis with surface acting specified as the final outcome, the interaction between SSR and sample membership was significant in the equation for internal employability (*b* = 0.30, *p* < 0.01). Furthermore, the conditional association between SSR and internal employability was positive in both samples but stronger for the Branch B sample (Branch A: *b* = 0.20, *p* < 0.01; Branch B: *b =* 0.49, *p <* 0.001).

In the surface acting equation, the interaction between SSR and sample membership was also significant (*b* = 0.32, *p* < 0.01). The conditional direct association between SSR and surface acting was negative in Branch A (*b* = −0.35, *p* < 0.001), but was not significant in Branch B (*b* = −0.03, *p* = 0.77). The interaction between internal employability and sample membership was significant as well (*b* = −0.38, *p* < 0.01). Internal employability was negatively associated with surface acting in Branch A (*b* = −0.18, *p* < 0.05) and Branch B (*b* = −0.56, *p* < 0.001). This negative association was stronger in Branch B.

The conditional indirect association between SSR and surface acting through internal employability was not statistically significant in the Branch A sample (indirect effect = −0.04, BootSE = 0.03, 95% BootCI [−0.10, 0.002]), but was significant and negative in the Branch B sample (indirect effect = −0.28, BootSE = 0.08, 95% BootCI [−0.43, −0.10]). The index of moderated mediation was negative and its bootstrap confidence interval excluded zero (index = −0.24, BootSE = 0.09, 95% BootCI [−0.40, −0.05]). The indirect association was significantly stronger in the Branch B sample than in the Branch A sample.

#### 4.7.2 Deep acting as the outcome

In the conditional process analysis with deep acting as the outcome, the interaction between SSR and sample membership was significant in the equation for internal employability (*b* = 0.30, *p* < 0.01). The positive association between SSR and internal employability was stronger in Branch B (*b* = 0.49, *p* < 0.001) than in Branch A (*b* = 0.20, *p* < 0.01).

In the equation for deep acting, the interaction between SSR and sample membership was not significant (*b* = −0.04, *p* = 0.73). The conditional direct association between SSR and deep acting was positive and significant in Branch A (*b* = 0.17, *p* < 0.05), but was not significant in Branch B (*b* = 0.13, *p* = 0.20). In contrast, the interaction between internal employability and sample membership was significant (*b* = 0.36, *p* < 0.05). The conditional association between internal employability and deep acting was positive in the Branch A sample (*b* = 0.31, *p* < 0.001) and even stronger in the Branch B sample (*b* = 0.67, *p* < 0.001).

The conditional indirect association between SSR and deep acting through internal employability was positive and statistically significant in both samples (Branch A: indirect effect = 0.06, BootSE = 0.04, 95% BootCI [0.01, 0.15]; Branch B: indirect effect = 0.33, BootSE = 0.10, 95% BootCI [0.11, 0.51]). The bootstrap estimate of the difference in conditional indirect associations was 0.27 (BootSE = 0.11, 95% BootCI [0.03, 0.46]), indicating that the estimated indirect association was significantly stronger in the Branch B sample.

These findings suggest that several estimated direct and indirect associations differed by branch. With branch membership confounded with organizational, geographic, demographic, and other unmeasured differences, the analysis cannot identify which contextual factors underlie these differences. The interaction findings should therefore be interpreted as supplementary, exploratory evidence rather than confirmation of moderation by any specific branch-level characteristic.

### 4.8 Summary of the hypothesis testing results

Table 12 summarizes the hypothesis testing results for the two branch samples. The main conclusions are based on the separate PROCESS Model 4 bootstrap analyses. The Monte Carlo analysis was used as a supplementary sensitivity check. The pooled PROCESS Model 59 analysis served as a supplementary exploratory analysis to test whether the estimated direct and indirect associations varied between the two branches.

**Table 12:** Summary of hypothesis testing results.

Hypothesis	Theoretical relationship	Branch A	Branch B	Pattern across branches
H1	SSR → internal employability	Supported	Supported	Supported in both branch samples
H2a	SSR → surface acting (total association)	Supported	Supported	Supported in both branch samples
H2b	SSR → deep acting (total association)	Supported	Supported	Supported in both branch samples
H3a	Internal employability → surface acting	Not supported	Supported	Supported only in Branch B
H3b	Internal employability → deep acting	Supported	Supported	Supported in both branch samples
H4a	SSR → internal employability → surface acting	Not supported	Supported	Supported only in Branch B
H4b	SSR → internal employability → deep acting	Supported	Supported	Supported in both branch samples

The conclusions for H1-H3 are based on the separate PROCESS Model 4 regression analyses, whereas the conclusions for H4a and H4b are based on the bootstrap confidence intervals. Monte Carlo simulation was used as a supplementary sensitivity analysis and produced conclusions consistent with the PROCESS bootstrap results. The final column summarizes the pattern of hypothesis support across the two samples and does not itself constitute a formal test of between-sample differences. Exploratory comparisons using PROCESS Model 59 are reported in Section 4.7.

In both branches, SSR was positively associated with internal employability and deep acting. SSR was negatively associated with surface acting. The indirect associations through internal employability were supported in Branch B for both surface acting and deep acting. In Branch A, the indirect association with deep acting was supported, whereas the indirect association with surface acting was not supported. The Monte Carlo results were consistent with the PROCESS bootstrap results.

## 5 Discussion

### 5.1 Summary and interpretation of the main findings

This study examined the relationships among SSR, internal employability, and emotional labor strategies in two supermarket samples in China. The findings generally support the relevance of SSR to front-line employees’ internal career perceptions and emotional regulation. Across both samples, SSR was positively associated with internal employability (*β* = 0.21 to 0.52, *p* < 0.05) and deep acting (*β* = 0.21 to 0.42, *p* < 0.05). In addition, it exhibited a significant negative association with surface acting (*β* = −0.43 to −0.36, *p* < 0.001). Internal employability was also positively associated with deep acting in both samples (the Branch A sample: *β* = 0.26, *p* < 0.01; the Branch B sample: *β* = 0.57, *p* < 0.001). However, its expected negative association with surface acting was not statistically supported in the Branch A sample (*β* = −0.17, *p* > 0.05). These patterns suggest that supervisory relationships and career perceptions are associated with front-line employees’ emotional regulation, although the link between internal employability and surface acting was not consistently supported across the two samples.

#### 5.1.1 Positive association between SSR and internal employability

The positive association between SSR and internal employability was supported in both samples (*β* = 0.21 to 0.52, *p* < 0.05). This pattern is consistent with the argument that employees draw on the quality of their relationship with their immediate supervisor when evaluating their continued value and prospects within the organization. Supervisors often control or influence access to work assignments, developmental feedback, recommendations, training, and information about internal opportunities. Employees may interpret these experiences as evidence that their competencies are recognized and that the organization remains willing to invest in them.

Attachment theory and sociometer theory offer complementary explanations for this link. From an attachment perspective, dependable and responsive supervisory relationships may make the immediate work environment appear more predictable and supportive, reinforcing employees’ sense of relational security. From a sociometer perspective, recognition, inclusion, and constructive treatment may communicate that employees are valued members of the workplace. Collectively, these perspectives suggest that relational cues embedded in SSR may convey both dependability and social acceptance, thereby informing employees’ broader career-related evaluations.

#### 5.1.2 Associations between SSR and emotional regulation strategies

SSR was negatively associated with surface acting (*β* = −0.43 to −0.36, *p* < 0.001) and positively associated with deep acting (*β* = 0.21 to 0.42, *p* < 0.05) in both samples. These findings are consistent with the view that supportive supervisory relationships are associated with less emotional distancing and greater engagement with service roles. Employees who feel recognized and supported may be more willing to invest the cognitive and emotional effort required to align their internal feelings with expected emotional displays (Grandey, 2000).

However, these results do not imply that SSR alone accounts for employees’ emotional labor strategies. Moreover, the cross-sectional design does not establish the direction of the observed relationships. SSR may be associated with employees’ emotional regulation strategies, but employees’ emotional experiences at work may also shape their perceptions of supervisory relationships. The findings should therefore be interpreted as evidence of association rather than causation.

#### 5.1.3 The differential role of internal employability and cross-sample variation

A central finding is that internal employability was more consistently associated with deep acting than with reduced surface acting. Internal employability was positively associated with deep acting in both samples (the Branch A sample: *β* = 0.26, *p* < 0.01; the Branch B sample: *β* = 0.57, *p* < 0.001). By contrast, its negative association with surface acting did not reach the conventional significance threshold in the Branch A sample (*β* = −0.17, *p* > 0.05). Correspondingly, the hypothesized indirect association between SSR and surface acting through internal employability was not supported in Branch A. The strength of the associations varied between the two samples. The associations involving deep acting received more consistent support, whereas those involving surface acting showed greater cross-sample variation.

Surface acting and deep acting are conceptually distinct strategies that employees may use independently or concurrently (Grandey, 2000; Diefendorff et al., 2005). The asymmetric findings therefore refine our understanding of the role of internal employability. Greater perceived internal employability appears to be more consistently associated with employees’ efforts to align their experienced and displayed emotions, but it is not necessarily associated with less reliance on surface acting. Thus, greater engagement in deep acting should not be interpreted as implying less surface acting. The comparative design highlights this theoretical asymmetry rather than merely showing that the two branches differ. A study of the Branch B sample alone would have supported the predicted association between internal employability and surface acting, whereas a study of the Branch A sample alone would not. Examining both settings therefore prevents either result from being overgeneralized and identifies this association as requiring further theoretical refinement and empirical examination.

Descriptively, employees in the Branch B sample reported higher mean levels of SSR, internal employability, and deep acting, whereas employees in the Branch A sample reported a higher mean level of surface acting. The smaller sample size in Branch A (*N* = 121), relative to Branch B (*N* = 166), may have made relatively small associations more difficult to detect. These descriptive and design differences provide relevant empirical context but do not explain the cross-sample variation. Because the study did not directly assess organizational practices or other contextual conditions, it cannot determine why the association between internal employability and surface acting differed between the two branches. This variation should therefore be interpreted cautiously and examined in future multi-site studies with larger, adequately powered samples.

#### 5.1.4 Indirect associations and robustness analysis

The indirect associations differed across the two branch samples. In the Branch B sample, SSR showed supported indirect associations with surface acting and deep acting through internal employability. In the Branch A sample, only the indirect association with deep acting was supported.

The Monte Carlo results were consistent with the PROCESS Model 4 bootstrap estimates. For surface acting, the indirect association was unsupported in Branch A but negative and supported in Branch B. For deep acting, positive indirect associations were supported in both branches. This consistency suggests that the conclusions were robust to the method used to construct the confidence intervals.

The Branch B findings should be interpreted alongside the modest reliability of the focal measures (Cronbach’s alpha and McDonald’s omega = 0.76–0.80). These values indicate acceptable internal consistency but lower measurement precision than in Branch A. Because PROCESS used observed composite scores, measurement error was not modeled and may have attenuated the estimated coefficients and indirect associations.

The exploratory Model 59 analyses provided further evidence of between-sample variation. The SSR × sample interaction was significant in the internal employability equation, and the conditional indirect associations differed significantly between the branches for both outcomes. The indirect association with surface acting was more negative in Branch B, whereas the indirect association with deep acting was more positive. However, these comparative findings are exploratory. The data do not identify the organizational, regional, or demographic conditions responsible for the observed variation. Given the cross-sectional design, the results should be interpreted as differences in estimated associations rather than evidence of causal or temporal processes.

#### 5.1.5 Exploratory demographic findings

The exploratory demographic findings also require restrained interpretation. In the Branch A sample, age and organizational tenure were negatively associated with SSR and internal employability. Older and longer-tenured employees in this sample tended to report less favorable supervisory relationships and weaker perceptions of their internal employment prospects. While these patterns may reflect perceived limitations in reskilling, development, or internal mobility opportunities, the study did not directly assess factors such as career stagnation, skill obsolescence, age-related anxiety, discrimination, or differential access to developmental resources.

In the Branch B sample, age and tenure showed no significant associations with the focal variables. However, this lack of statistical significance should not be interpreted as evidence that formal human resource management practices effectively safeguarded older employees, nor that organizational resources were distributed independently of demographic characteristics. The observed cross sample differences should not be taken as supporting conclusions of demographic disadvantage in one context or institutional protection in another. Instead, they simply highlight questions that remain to be addressed, rather than serving as confirmatory evidence of divergent organizational outcomes.

#### 5.1.6 Summary of key findings

Overall, SSR and internal employability emerged as related but distinct resources in front-line retail work. Across both branch samples, higher quality SSR was associated with stronger internal employability, lower surface acting, and greater deep acting. Internal employability was more consistently associated with deep acting than with surface acting. In the Branch B sample, both hypothesized indirect associations through internal employability were supported. In the Branch A sample, the indirect association with surface acting was unsupported, whereas the indirect association with deep acting was supported.

### 5.2 Theoretical contributions

This study makes three main theoretical contributions to research on supervisory relationships, internal employability, emotional labor, and comparative management.

First, the study demonstrates how attachment theory and sociometer theory can serve as complementary interpretive lenses for understanding the relational sources of internal employability. Attachment theory addresses the security-related meaning of supervisory treatment, whereas sociometer theory addresses its evaluative meaning for employees’ perceived organizational standing. These perspectives therefore complement rather than compete, and their integration is interpretive rather than empirical.

The contribution does not claim that internal employability functions as an operational proxy for attachment security or as a direct sociometer reading. Instead, internal employability remains an independently measured career-related perception concerning employees’ confidence in retaining their current positions or obtaining alternative roles within the organization. The value of the framework lies in showing how supervisory experiences, when interpreted through both security and evaluative lenses, may acquire broader career-related meaning, rather than in identifying two distinct psychological transmission mechanisms.

These complementary perspectives also clarify the conceptual boundary between SSR and internal employability. SSR concerns the present quality of a specific dyadic relationship, whereas internal employability concerns anticipated continuity and mobility within the broader organization. The two constructs may be associated because employees use supervisory experiences as career-related information, but the relationship is probabilistic rather than definitional. By acknowledging that attachment and sociometer processes were not directly measured, the study positions these perspectives as interpretive frames that enrich the understanding of observed associations rather than as competing explanations to be empirically tested.

Second, the study extends research on internal employability by demonstrating its differential associations with emotional labor strategies. Existing employability research has largely focused on competencies, adaptability, career development, and labor-market opportunities (Nauta et al., 2009; Akkermans et al., 2019; De Vos et al., 2021; Wilhelm et al., 2024). The present findings suggest that employees’ internal employability perceptions may also be informed by relational information embedded in routine supervisory experiences.

The findings further indicate that internal employability does not relate symmetrically to surface acting and deep acting. Its positive association with deep acting was supported in both samples, whereas its negative association with surface acting was not supported in the Branch A sample. This pattern is consistent with the view that deep acting and surface acting are distinct emotional labor strategies that may relate differently to career-related resources (Diefendorff et al., 2005).

Employees who perceive greater internal employability may anticipate greater value from emotional investment and thus be more willing to expend effort on deep acting. By contrast, the predicted negative association between internal employability and surface acting was not consistently supported across the two samples. The theoretical contribution therefore lies in showing that internal employability is differently associated with these distinct emotional labor strategies. It was more consistently associated with greater engagement in deep acting than with reduced reliance on surface acting. This finding suggests that the prediction for H3a derived from sociometer theory requires further theoretical refinement and empirical testing.

Third, the study offers exploratory comparative evidence across two organizational settings. The data do not establish whether the observed differences reflect ownership, institutional context, or other unmeasured characteristics. Configural, metric, and scalar invariance held across samples, supporting meaningful comparisons of the estimated relationships. The findings revealed shared patterns across the samples, including positive associations between SSR and internal employability and between internal employability and deep acting. By contrast, the results for relationships involving surface acting and the indirect effects were less consistent across the samples. This comparison highlights similarities and differences in the observed associations across the two branches and motivates further research on possible contextual boundary conditions.

These variations illustrate the value of testing the same theoretical model across multiple settings. A single branch study would have yielded different conclusions depending on which branch was selected. By incorporating two samples, the present design provides a more comprehensive empirical overview and reduces the risk of overgeneralizing from a single workplace. However, its contribution does not lie in establishing a fixed distinction between institutional systems, as sample membership coincided with a combination of organizational, regional, and demographic differences. The findings suggest that the observed relationships may not generalize uniformly across settings, although the available data do not allow us to identify the specific conditions driving this variation. Accordingly, the comparative evidence should be viewed as exploratory and suggestive rather than conclusive.

### 5.3 Practical implications

The findings suggest that relational support, internal career perceptions, and emotional regulation may be usefully managed as connected but distinct aspects of front-line retail work. Because the present study did not directly evaluate specific managerial interventions, the following recommendations should be understood as tentative implications derived from the observed associations rather than as demonstrated intervention effects.

First, retail organizations may consider strengthening the supportive and developmental aspects of front-line supervision. Supervisors can serve not only as operational coordinators but also as sources of interpersonal support, developmental information, and assistance during difficult service encounters. Regular supervisor-employee conversations could address available support, emotionally demanding customer interactions, perceived skill relevance, and realistic internal opportunities. Discussing these topics separately may help managers distinguish relational concerns from career uncertainty and situational emotional demands.

Following difficult customer incidents, timely supervisory support may also be beneficial. Structured discussions could examine what occurred, the emotional effort involved, whether assistance or escalation was available, and what forms of operational or developmental support might reduce similar strain. Such discussions should not assume that employees’ displayed emotions provide a direct indication of sincerity or commitment. Surface acting should not automatically be interpreted as evidence of low sincerity or weak organizational commitment.

Supervisory feedback could also be combined with customer-conflict coaching, clearer escalation procedures, temporary task adjustment, guided practice, or relevant training. Although these practices were not directly examined in this study, they may help employees manage difficult interactions and clarify the support available to them.

Second, organizations may benefit from distinguishing discussions of internal employability from conventional performance appraisal. A supportive supervisory relationship does not necessarily ensure that employees feel confident about their future prospects. Whereas performance appraisal typically concerns current or past performance, an internal employability discussion could focus on future skill relevance, employment continuity, and internal mobility.

Such discussions may clarify current competencies, changing job requirements, development needs, and realistic opportunities for training, role expansion, transfer, or promotion. Where appropriate, they could lead to development plans specifying the responsibilities of employees, supervisors, and the organization. Managers should nevertheless communicate realistic possibilities rather than unsupported assurances of promotion or continued employment.

In more formalized organizations, supervisors may need to help employees interpret competency frameworks, digital vacancy systems, and standardized appraisal criteria. General criteria could be linked to observable behaviors, available development opportunities, and the evidence required for progression. Transparent procedures may also be useful when employees encounter disputed evaluations, inaccessible training, or unclear promotion criteria.

Third, career support should not be treated solely as a means of reducing surface acting. In the present study, internal employability was more consistently associated with greater deep acting than with lower surface acting. Managers should therefore avoid assuming that efforts to enhance employees’ internal employability will necessarily reduce their reliance on surface acting. Such efforts may be more appropriately viewed as having the potential to encourage deep acting, although this practical implication requires further evaluation through longitudinal and intervention studies.

The exploratory demographic findings also suggest that organizations may benefit from examining whether supervisory and developmental resources are distributed consistently across employee groups. Access to feedback, training, reskilling, career discussions, internal vacancies, transfers, and promotions could be reviewed by age and tenure. Any observed differences should prompt further investigation rather than immediate conclusions about discrimination. Confidential surveys, interviews, or focus groups may help clarify the processes underlying such patterns.

Brief internal employability surveys could also be used to assess employees’ confidence in retaining their roles, qualifying for alternative positions, maintaining skill relevance, and accessing developmental support. Such information would be most appropriately interpreted at an aggregate level and should not be used to penalize employees who report lower career confidence.

Digital transformation creates additional issues that extend beyond the variables directly examined in this study (Mendy et al., 2024). When automated scheduling, self-checkout technologies, AI-assisted monitoring, or service robots are introduced, organizations may consider providing clear information about task changes, relevant competencies, training access, and decision criteria. These suggestions are inferential, but they are consistent with the broader finding that employees’ career-related perceptions may depend partly on the information and support available within the organization.

Where algorithmic monitoring is used, transparency and human oversight may also warrant consideration. Organizations could explain what data are collected, how automated outputs inform scheduling or evaluation, who reviews those outputs, and how inaccurate information may be challenged. Customer ratings, facial-expression analysis, voice data, and productivity indicators should not automatically be treated as definitive measures of sincerity, emotional commitment, or internal employability. These recommendations represent broader governance considerations rather than practices directly tested in the present study.

## 6 Limitations and future research

The findings should be interpreted in light of several limitations. First, the cross-sectional design does not establish temporal order or causality. The indirect effect results indicate statistical associations rather than confirmed psychological mechanisms, and reverse or reciprocal relationships remain possible. Longitudinal, cross-lagged, diary, and field experimental studies are needed to examine temporal sequencing and within-person change.

Second, all focal variables were self-reported by front-line employees at a single time point, creating risks of CMV, social desirability, and consistency related responding. Harman’s single-factor test does not demonstrate the absence of method bias, particularly because the first-factor variance in Branch A was not negligible. Future studies should incorporate temporal separation, marker variables, multi-source data, and objective indicators.

Third, the comparison between the two branch samples is vulnerable to contextual confounding and limited generalizability. Data were collected from one Shenzhen branch of a local private supermarket chain and one Beijing branch of a Western multinational chain. The samples differed in organizational background, geographic location, workforce composition, labor market conditions, and potentially other unobserved practices. Sample membership should therefore not be interpreted as a pure indicator of ownership or institutional context, and the comparison should be viewed as exploratory rather than as a formal test of organizational moderation.

In addition, both branches were located in first-tier Chinese cities and represented only the supermarket sector, limiting generalizability to other regions, city tiers, organizational settings, industries, and service occupations. Future research should include multiple branches, organizations, regions, and occupational settings, using matched-sample, multi-site, and multilevel designs together with direct measures of organizational conditions. Studies conducted in lower-tier cities, less developed regions, or other countries would also require renewed measurement-invariance testing and careful consideration of cultural and institutional differences.

Fourth, the focal measures in the Branch B sample demonstrated acceptable but comparatively modest reliability. Given that PROCESS relied on observed composite scores, measurement error was not explicitly accounted for and may have affected the magnitude and precision of the estimated regression coefficients and indirect associations. Future research should use latent-variable mediation models and investigate the sources of reliability differences across samples.

Fifth, attachment theory and sociometer theory were used as complementary interpretive perspectives, but the proposed psychological processes were not directly measured. Future studies should assess attachment security, relational dependability, perceived acceptance, organizational inclusion, and sociometric appraisal. Their relative explanatory value for the relationship between SSR and internal employability should also be compared. Research should further examine the cognitive and motivational processes linking internal employability to emotional labor, including anticipated returns from emotional investment, resource conservation, engagement, emotional risk, and self-protection.

Sixth, although the PROCESS and Monte Carlo analyses produced consistent conclusions for all four indirect associations, the estimates for the Branch A sample were comparatively imprecise. In particular, the lower confidence limits for the indirect association with deep acting were close to zero, whereas the confidence intervals for the indirect association with surface acting included zero. Replication using larger samples, prospective power analyses tailored to indirect associations, preregistered analyses, and latent variable models is therefore needed.

Seventh, the demographic findings were exploratory. The study did not directly assess age discrimination, career plateauing, skill obsolescence, access to reskilling, or fairness in developmental opportunities. Future research should distinguish among age, organizational tenure, career stage, occupational experience, and employment status when examining the distribution of supervisory and career-related resources.

Finally, future research should examine how digital transformation reshapes SSR, internal employability, and emotional labor. Automated scheduling, AI-assisted monitoring, customer rating systems, self-checkout technologies, and service robots may alter employees’ access to supervisory support and their perceptions of future organizational value. Studies should distinguish developmental from punitive monitoring and assess transparency, human review, reskilling opportunities, and appeal procedures. Longitudinal research conducted before, during, and after technological implementation, combining employee surveys with organizational records, would provide stronger evidence of how these relationships operate in increasingly digitized retail workplaces.

## 7 Conclusion

This study examined how SSR and internal employability relate to front-line employees’ emotional labor in two Chinese supermarket samples. Across both samples, higher-quality SSR was associated with stronger internal employability, lower surface acting, and greater deep acting. Internal employability was positively associated with deep acting in both samples, whereas its negative association with surface acting was supported only in the Branch B sample.

The indirect effect results differed across the branch samples. Both hypothesized indirect associations were supported in Branch B. In Branch A, the indirect association involving surface acting was unsupported, whereas the indirect association involving deep acting was supported. These findings represent sample-specific patterns rather than effects attributable to ownership or institutional context.

Overall, internal employability emerged as a distinct career-related link between SSR and emotional labor. Attachment theory and sociometer theory provide complementary interpretations of these associations but were not tested as separate mechanisms. Future longitudinal, multi-source, multi-site, and latent-variable research should further examine the organizational and technological conditions shaping these relationships.

## Data Availability

The raw data supporting the conclusions of this article will be made available by the authors, without undue reservation.
